# Isolation and Characterization of “Terrein” an Antimicrobial and Antitumor Compound from Endophytic Fungus *Aspergillus terreus* (JAS-2) Associated from *Achyranthus aspera* Varanasi, India

**DOI:** 10.3389/fmicb.2017.01334

**Published:** 2017-07-25

**Authors:** Jyoti Goutam, Gunjan Sharma, Vinod K. Tiwari, Amrita Mishra, Ravindra N. Kharwar, Vijayakumar Ramaraj, Biplob Koch

**Affiliations:** ^1^Department of Botany, Institute of Science, Banaras Hindu University Varanasi, India; ^2^Department of Zoology, Institute of Science, Banaras Hindu University Varanasi, India; ^3^Department of Chemistry, Institute of Science, Banaras Hindu University Varanasi, India; ^4^Department of Microbiology, Sri Ramachandra University Porur, India

**Keywords:** environmental mycology, chromatography, anticancer activity, Terrein, lung cell line (A-549), anti-bacterial agents, antifungal activity

## Abstract

The present study aimed at characterizing biological potentials of endophyte *Aspergillus terreus* JAS-2 isolated from *Achyranthus aspera*. Crude extracted from endophytic fungus JAS-2 was purified and chemically characterized by chromatographic and spectroscopic studies respectively. Spectral assignment of NMR (nuclear magnetic resonance) data, ^1^H proton and ^13^C carbon analysis along with FTIR data elucidated the structure of compound as 4,5-Dihydroxy-3-(1-propenyl)-2-cyclopenten-1-one. After purification and identification a set of experiment was conducted to explore efficacy of compound. Results revealed that on accessing the antifungal activity of compound, growth diameter of tested phytopathogenic fungi was reduced to 50% at higher concentration taken (10 μgμl^−1^). Compound exhibited *in-vitro* bacterial cell inhibition at 20 μgml^−1^ concentration along with moderate antioxidant behavior. Evaluation of anticancer activity against human lung cancer cell line (A-549) exhibited its IC_50_ value to be 121.9 ± 4.821 μgml^−1^. Further cell cycle phase distribution were analyzed on the basis of DNA content and evaluated by FACS (Fluorescence Activated Cell Sorting) and it was revealed that at 150 μgml^−1^ of compound maximum cells were found in sub G1 phase which represents apoptotic dead cells. Terrein (4, 5-Dihydroxy-3-(1-propenyl)-2-cyclopenten-1-one) a multi-potential was isolated from endophytic fungus JAS-2, from well recognized medicinal herb *A. aspera*. To best of our knowledge, this is the first report of “Terrein” from endophytic derived fungus. This compound had also exhibited anticancer and antifungal activity against human lung cancer cell line A-549 and *Bipolaris sorokiniana* respectively which is causal organism of many plants disease. Hence endophytes are serving as alternative sources of drug molecules.

## Introduction

Endophytic microbial are reputed entities for their rich metabolic factories. They colonize inter and intracellular spaces of tissues of higher plants without causing apparent damage to the plants in which they live. Till now, numerous researches have been conducted on structure and functions of endophytic bioactive natural products (Schulz et al., 2002; Molina et al., 2012). Previous studies indicated them as potent herbicides, insecticides (Hu et al., 2008), antimicrobials (Zhang et al., 2013; Goutam et al., 2016a), and antimalarial compounds (Zaher et al., 2015). However, there are several chronic disorders like cancer, diabetes, and infectious diseases for which cure/treatment need to be enhanced (Costello et al., 2012). Hence, investigation on endophytic fungi for bioactive compounds is important for finding cure for several disease/disorders.

Despite the substantial work on endophytes, much of microbial diversity of nature remains to be explored; it was declared that ~61% of natural drugs are used in the areas of infectious diseases and cancer (Newman and Cragg, 2007).

Terrein a secondary metabolite was first described from fungal strain by Raistrick and Smith (1935). It was reported for various metabolite strength such as hypopigmentary effect (Park et al., 2004) and anticancer properties. And the role of Terrein in contributing to the medicinal value of fungal products has already been documented in literatures. Herein we have demonstrated identification and characterization of bioactive compound Terrein isolated from endophytic fungus *Aspergillus terreus* JAS-2 associated with medicinal plant *Achyranthus aspera*. The compound characterized and confirmed as 4, 5- Dihydroxy-3- (1-propenyl) -2-cyclopenten-1-one (Terrein) Terrein had shown good anticancer and antifungal activities. Furthermore, moderate antibacterial and antioxidant properties are also reported.

## Materials and methods

### Endophytic strain *Aspergillus terreus* (JAS-2)

The culture was obtained from medicinal plant *A. aspera* (Prickly chaff flower). It was identified as *A. terreus* on the basis of its macroscopic and microscopic characteristics (Goutam et al., 2016b). Culture was maintained on potato dextrose agar (PDA) at 4°C and sub cultured every 15 days. The culture was grown in sabouraud's dextrose broth (SDB) and sabouraud's dextrose agar (SDA) for various activities.

### Column chromatography (CC) (SiO_2_) and thin layer chromatography (TLC)

Column chromatography (SiO_2_) was performed in a glass column (700 ×30 mm). Silica gel (100–120 mess size Merck) was used as stationary phase. Mobile phase consist of pure solvent or different solvents depending upon requirements. Column was loaded with crude extract from *A. terreus* (JAS-2). Mobile phase consist of methanol and chloroform in 5:95, 10:90, ratio was used for separating compound and gradient elution was followed. Different fractions eluted from column chromatography (CC) were separated by thin layer chromatography (TLC) and assessed for antibacterial activity.

### Analytical thin layer chromatography (TLC)

TLC was performed on 60F_254_ silica gel, pre-coated on aluminum plates (Merck). Eluted solvents were evaporated under reduced pressure on *IKA Rotary Evaporator* at temperature <50°C. From each fraction 10 μgμl^−1^ of compound was spotted with fine capillary tubes, using Methanol and chloroform (10:90) as mobile phase and the spots were identified in UV Chamber.

### Nuclear magnetic resonance spectroscopy

Extensive spectroscopic data (^1^H and ^13^CNMR) were recorded on *JEOL AL300 FT-NMR Spectrometer* at 300 and 75 MHz, respectively. Chemical shifts given in ppm downfield from tetramethylsilane TMS (δ = 0.0 ppm) as internal reference; *J*-values in Hz. Solvents were D_2_O and DMSO-d_6_.

### FTIR (fourier transformer infrared spectroscopy)

Infrared spectra recorded as Nujol mulls in KBr plates. IR spectral data was recorded on FTIR-8201 PC Shimadzu spectrometer.

### GCMS (gas chromatography mass spectroscopy)

GC-MS analysis was carried out in GCMS QP 2010 plus shimadzu ultra-gas chromatograph coupled with a series mass selective detector. Pressure was kept 90.4 kpa. The column temperature was 100°C initially with a hold of 3 min, then programmed to 280°C at a rate of 5°C/min for 5 min and programmed to 320°C for 19 min at the rate of 10 min/s. Helium was used as the carrier gas and the column head pressure was maintained at 13.3 psi. Injector temperature was maintained at 260°C, and the injection volume was 1.21 ml min^−1^ in the split mode. And interface temperature was held at 270°C. Mass spectra were scanned from *m*/*z* 40 to 550 with a scan speed of 3,333. Initial identification of crude complex and purified compound was done by comparison of compound with the National institute of standard and Technology (NIST) database.

### Minimum inhibitory concentration (MIC) (IC_50_ value) against *Staphylococcus aureus, Aeromonas hydrophila*, and *Enterococcus faecalis*

The MIC of purified compound against pathogens was determined by MTT method with minor modification (Jorgensen and Ferraro, 2009). Compound was dissolved in DMSO maintaining a stock concentration of 1 μgμl^−1^. The compounds from 2 to 20 μgμl^−1^ concentrations were taken along with 5 μl overnight bacterium inoculums (log phase) and dispensed into individual vial. Mueller Hinton Broth (MHB) was added to maintain the concentration from 2 μgml^−1^ to 20 μgml^−1^. Subsequently the vial sets were incubated overnight at 37 ± 2°C. On next day 60 μl of 5 mgml^−1^ MTT (3-(4, 5-Dimethylthiazol-2-yl)-2, 5-Diphenyltetrazolium Bromide) (Sigma, USA) dissolved in PBS was dispense in each well to examine the microbial growth. All vials were centrifuged at 10,000 × g for 5 min and absorbance was measured at 570 nm using Spectrophotometer. The procedure was repeated thrice to check the reproducibility. The lowest concentration of test compound at which no growth was seen considered as MIC. The absorbance for each well was calculated as: Ab (570 nm) of the sample–Ab (570 nm) of the control; Where the control contains only the culture medium and test compound solvent. The results were expressed in μgml^−1^.

### Calculation of % cell viability

(1) $$\begin{array}{r} \frac{OD\;of\;Bacterial\;Growth\;\left(C\right)-OD\;of\;Bacterial\;growth\;with\;test\;sample\times 100}{OD\;of\;Bacterial\;growth\;\left(\;c\right)Control}\;=\%\;Cell\;viability \end{array}$$

### *In vitro* antifungal test by linear mycelia growth reduction assay

Pure compound separated from CC was used for antifungal test against pathogenic fungi such as *Alternaria alternata, Bipolaris sorokiniana, Phytopthora drechsleri*, and *Aspergillus flavus*. The assay was performed using pour plate method according to Ranware et al. (2010).

Briefly, four different concentration of compound was prepared with DMSO (100, 200, 500, and 1,000 mgml^−1^). One milliliter of each concentration of compound was mixed to 99 ml of pre-sterilized potato dextrose agar media (50°C). On the following day equal size of fungal plug of all pathogenic were inoculated in plate having stock concentration ranges from 1, 2, 3, 5, and 10 μgμl^−1^. Inoculations of all the four fungal isolates were done separately. Media with only plain DMSO was used as negative growth control. The plates were observed for 5–10 days for reduction in fungal growth by measuring the radius of growth in both control and test plate. All the concentrations were tested in triplicates for all the pathogens to ensure the reproducibility of the results.

(2) $$\begin{array}{r} \frac{Fungal\;growth\;\left(C\right)-Fungal\;growth\;of\;treatment\times 100}{Fungal\;growth\;\left(c\right)\;Control}\;=\%\;Reduction\;in\;fungal\;growth \end{array}$$

### *In-vitro* antioxidant activity by DPPH (2, 2-Diphenyl-1-Picrylhydrazyl) scavenging activity of compound (IC_50_)

*In vitro* antioxidant activity of compound was performed using standard methodology of Marwah et al. (2007). Different concentration of compound was prepared from 10 to 100 μg in methanol. All tests were carried out in triplicates. The percentage of free radical scavenging activity of compound was calculated as % I = [(A _blank_ − A _sample_)/A _blank_] ×100 Where A _blank =_ Absorbance of control, A _sample_ = Absorbance of methanolic extract and DPPH, IC_50_ was calculated by plotting straight line equation.

### Assessment of anticancer activity of crude and isolated compound 1

#### Lung cancer cell line

Lung cancer cell line (A-549) was obtained from National Centre for Cell Science (NCCS), Pune, India. The cells were maintained in Dulbecco Modified Eagle Medium (DMEM) with L-glutamine supplemented with 10% fetal bovine serum (FBS) and penicillin, streptomycin and incubated in 5% CO_2_ condition at 37°C. The cells were harvested when they reached 80–90% confluence and plated for subsequent experiments.

#### Cell viability and proliferation assay

The crude extract and purified compound were screened for cytotoxicity and anti-proliferative activity by MTT assay (Mossman, 1983). Stock solutions of compounds were prepared in DMSO (50 μgμl^−1^) and further diluted in complete DMEM (DMEM with 10% FBS). A-549 cells (1 × 10^4^ cells/well) were seeded in 96-well plate and 4 replica wells were used for controls as well as treated groups. After 24 h of incubation, culture medium was replaced by fresh medium containing various concentrations of extracts (10–300 μgml^−1^) and incubated for 24 h with 5% CO_2_. After incubation, MTT assay was performed as reported earlier (Paul et al., 2014).

Fifty percentage inhibitory concentration (IC_50_) values of both extracts were calculated as the percentage of treated cells relative to control cells. The percent of the cell viability was calculated using the equation: (2)

(Mean O.D. of treated cells/mean O.D. of control cells) X 100

### Analysis of nuclear morphology with hoechst and propidium iodide (PI) staining after drug exposure

Chromatin condensation and fragmentation of the nucleus can be seen with fluorescence microscopy after staining with DNA binding fluorescence dyes.

#### Staining procedure

A-549 cells were seeded in six-well plates and treated with different concentrations including IC_50_ value of both extracts for 24 h in CO_2_ incubator. Subsequently, cells were washed with PBS and stained with Hoechst (10 μgml^−1^) and Propidium Iodide (10 μgml^−1^). After staining, cells were washed with PBS and imaged by inverted fluorescent microscope (EVOS FL Cell Imaging System, Thermo Fisher Scientific) in phase contrast, blue and red channels.

#### Cell cycle analysis by fluorescence activated cell sorting (FACS)

Apoptosis is characterized by DNA fragmentation and consequently loss of nuclear DNA content of the cells. The DNA content during cell cycle steps was evaluated by flow cytometry with PI (Riccardi and Nicoletti, 2006).

#### Treatment and sample preparation

A-549 cells (1 × 10^5^ cells/well) were treated with three different concentrations (near to its IC_50_ value) of crude and pure compound Terrein for 24 h in complete DMEM. After 24 h, cells were stained with PI and analyzed by FACS (BD Biosciences) as described previously (Singh et al., 2016).

## Results

### Isolation and purification of compound 1

#### Column chromatography (CC) and thin layer chromatography (TLC)

**Figure 1:**
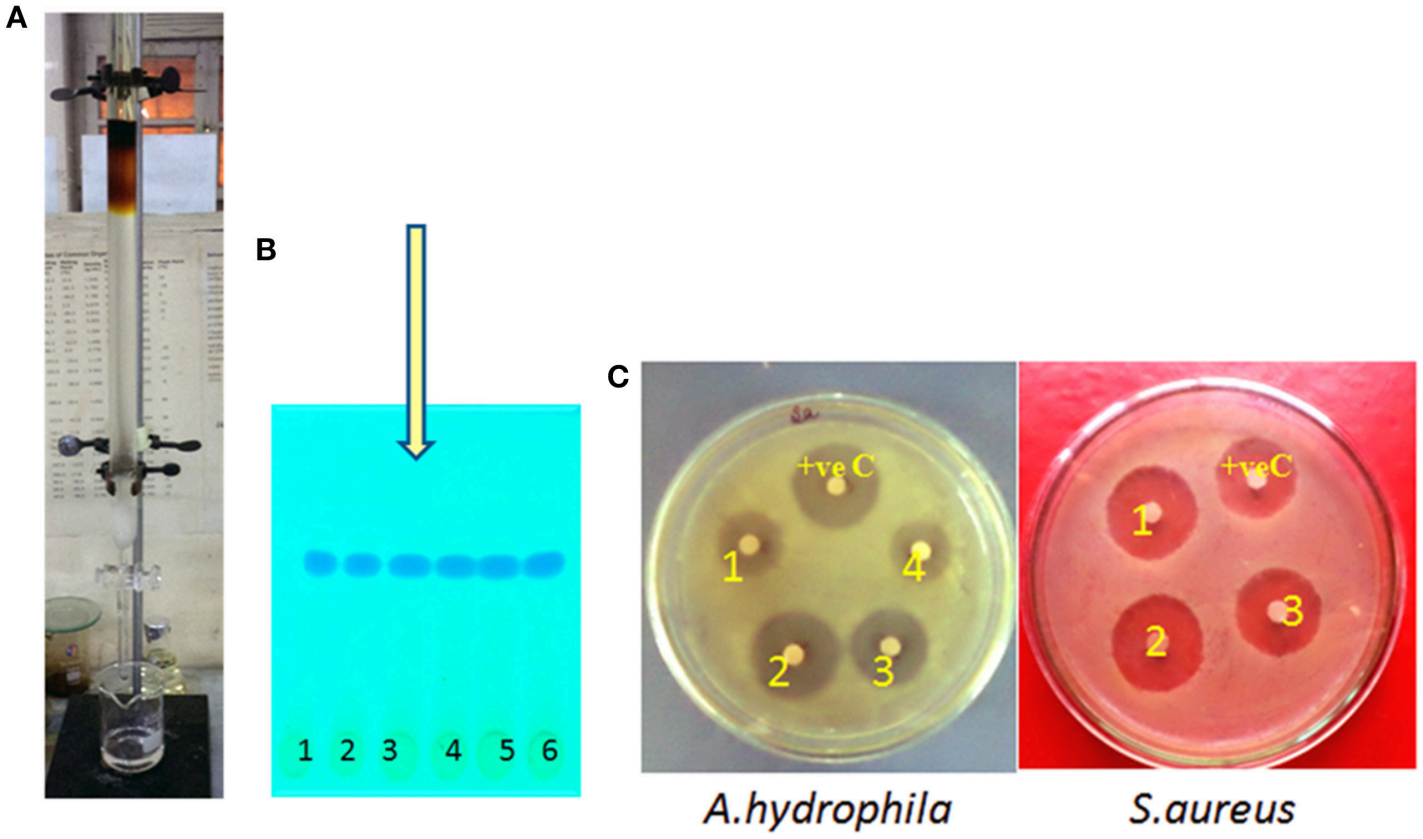
**(A)** Separation of pure compound from silica gel column chromatography (CC), **(B)** thin layer chromatography (TLC) and its **(C)** assessment for antibacterial activity.

The crude isolated from JAS-2 was directly chromatographed using silica gel column eluted with a CHCl3/MeOH gradient (Figure 1A). Total of 25 fractions of 20 ml were collected carrying retention time of 5 ml min^−1^. All fraction collected was subjected to TLC analysis (Figure 1B) and single spot was observed. The retention time was at 0.58 (Figure 1B). The fraction exhibited antibacterial activity against *S aureus* and *A hydrophila* (Figure 1C).

#### One dimensional NMR (proton and carbon NMR) and IR of compound

**Figure 2:**
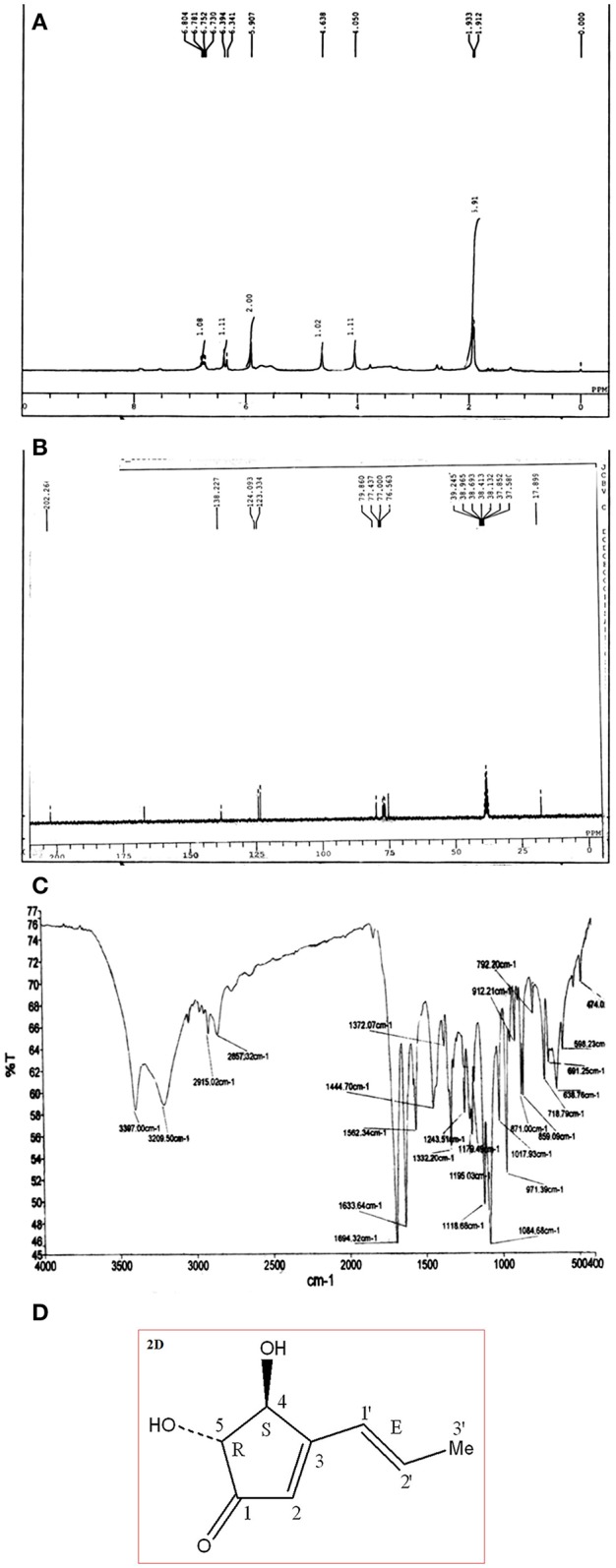
**(A)** Proton ^1^H NMR of pure compound separated via CC and TLC in Figure 1. **(B)** ^13^C NMR of pure compound separated via CC and TLC in Figure 1. **(C)** FTIR of pure compound Terrein separated via CC and TLC in Figure 1. **(D)** Structure of characterized compound Terrein (Figure 1).

The compound characterization was carried out using proton and carbon NMR. 1H NMR (300 MHz, CDCl3) A triplet integrated to three protons at δ 1.92 evidenced the presence of a methyl group. Appearance of two broad singlets' at δ 4.63 and 4.06 integrated to one proton each showed the presence of two hydroxyl hydrogen's. Two multiplet at δ 5.90 and 6.36; integration of two proton each; showed the presence of two alkenic and two methylene protons at C1 carbon (adjacent to carbonyl group). A multiplet of 2 protons appeared at δ 6.76 which corresponds to the two–CH proton present at the hydroxyl group carbon. All above details of proton environment can be collectively analyzed by Figure 2A. ^13^C NMR (75 MHz, DMSO): δ 167.1, 138.2, 124.0, 123.3, 79.8, 78.7, 75.8, 17.8 ppm (Figure 2B) IR (KBr) ν_max_: 3,397; 2,915, 2,857, 1,694 (C = O), 1,332, 1,118 cm^−1^ (Figure 2C). Compound **1** was characterized as 4, 5-Dihydroxy-3-(1-propenyl)-2-cyclopenten-1-one, trade name of compound is Terrein. (Figure 2D).

### GCMS (gas chromatography mass spectroscopy)

Crude extracted from PDB and pure compound from column chromatography (CC) and thin layer chromatography (TLC) were subjected to GCMS analysis. Based on MS library of NIST and WILEY, single peak was detected from pure compound (Figure S1A) whereas multiple peaks were detected from crude extracted (Figure S1B). All major peaks resolved through GCMS and presented in the chromatogram (Figures S1A,B).

Total 14 peaks were observed from the chromatogram of crude extract. **Tables 4, 5** shows number of peaks, retention time, area (%), and matching factor of compound present. In **Table 5** only one compound was showing 100% identity with Terrein, whereas in **Table 5** compound showing only 91.85% identity as other compounds present in crude extract.

### Estimation of antimicrobial and antioxidant activity of compound

**Figure 3:**
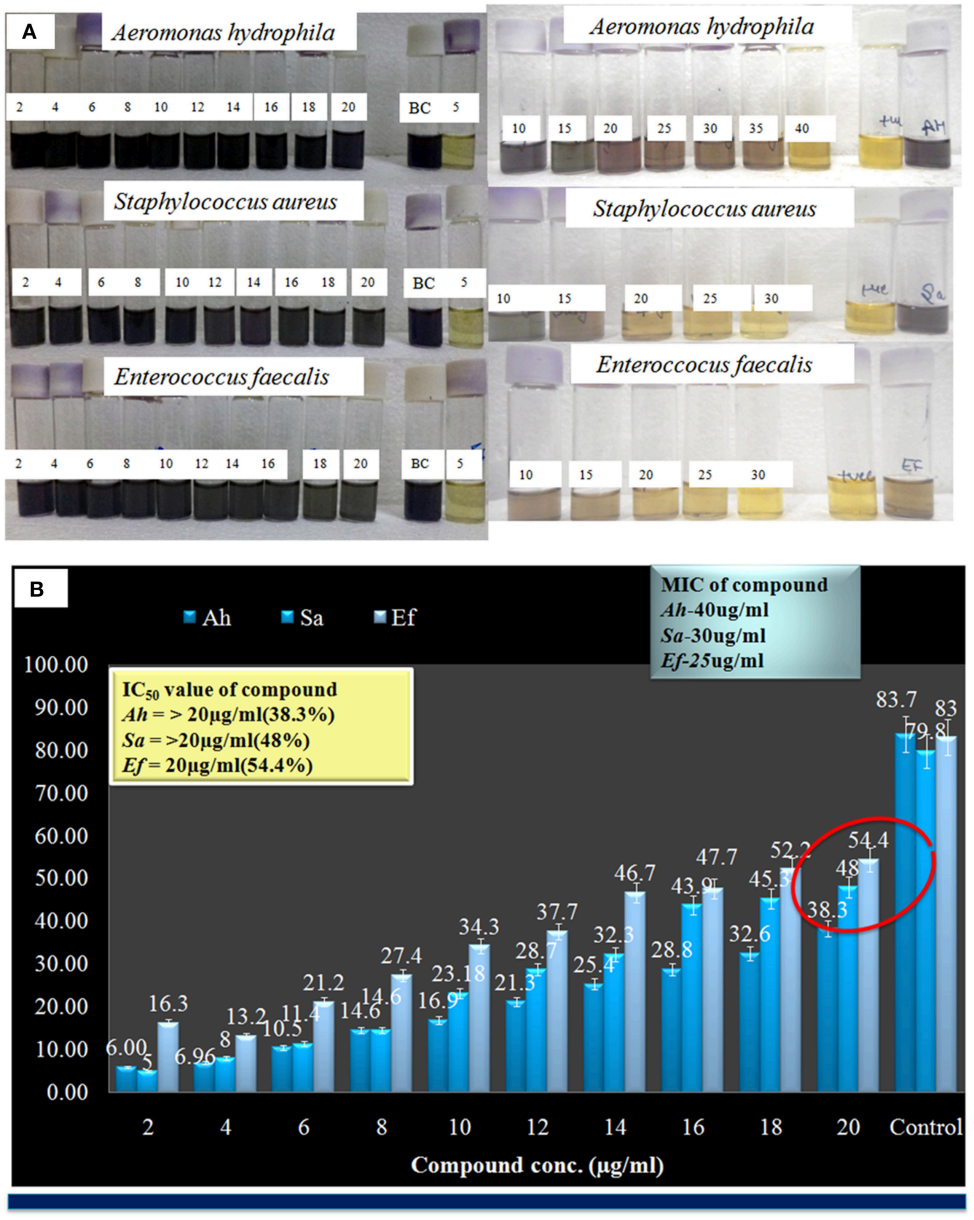
**(A)** Evaluation of minimum inhibitory concentration (MIC) Terrein via MTT assay. **(B)** Graphical presentation of IC-50 values of Terrein.

**Figure 4:**
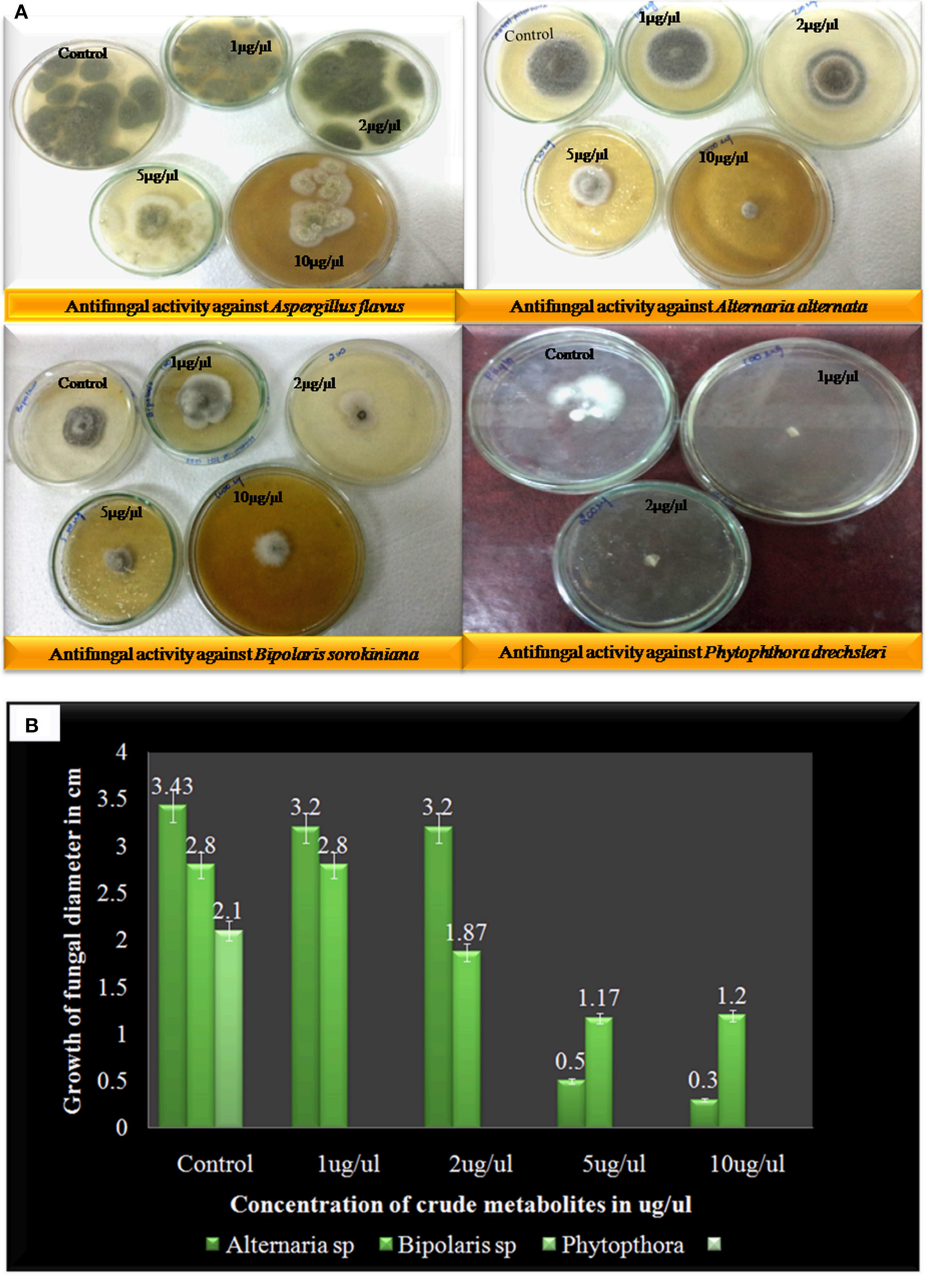
**(A)** Antifungal activity of Terrein against different plant fungal plant pathogens via linear reduction mycelia assay. **(B)** Graphical presentation of pathogenic fungal inhibition test (2, 2-Diphenyl-1-picrylhydrazyl) via Terrein.

**Figure 5:**
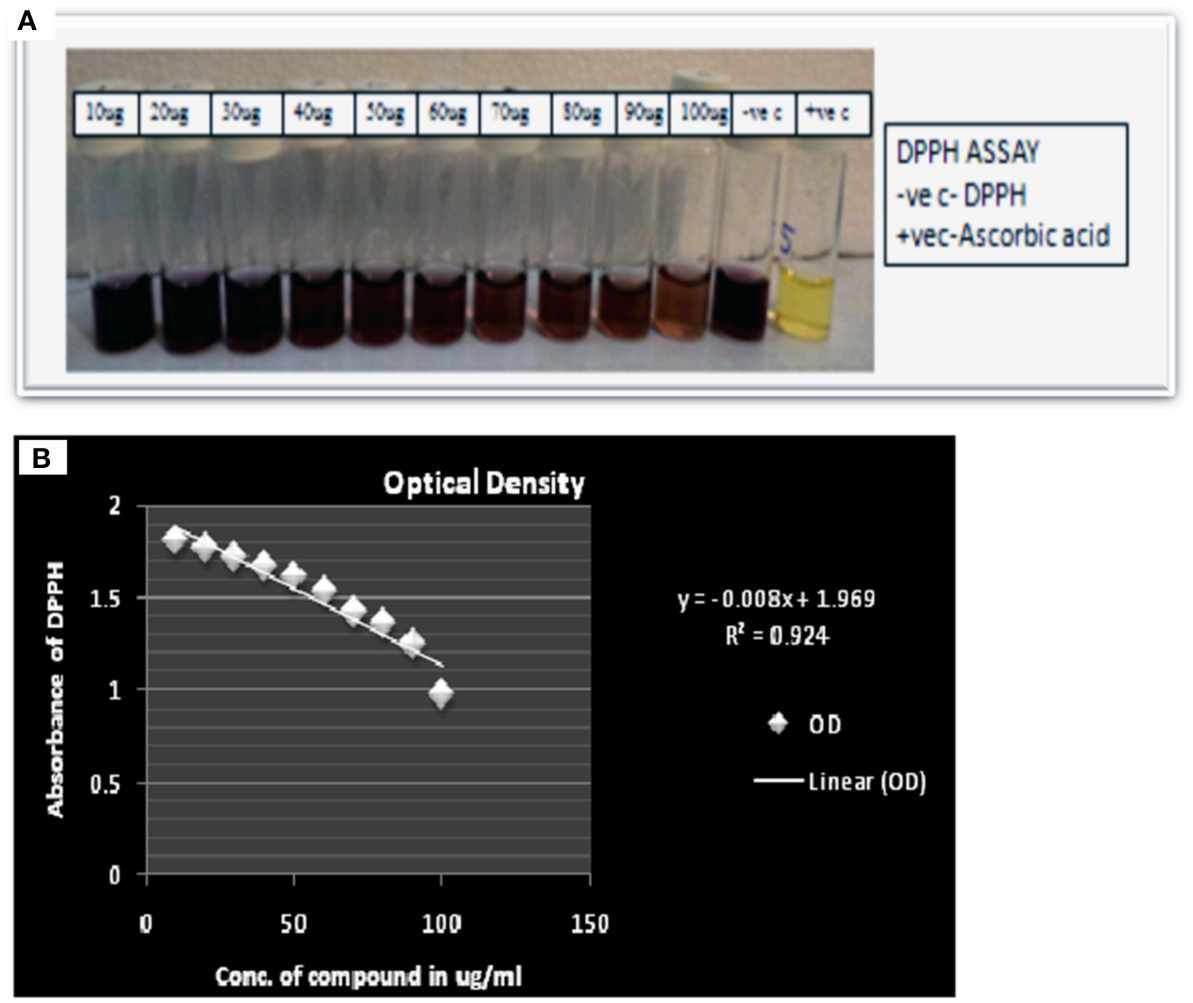
**(A)** Scavenging activity via DPPH assay of Terrein. **(B)** Linear regression curve of Terrein showing concentration verses absorbance in (2, 2-Diphenyl-1-picrylhydrazyl) DPPH assay.

Here compound purified from extract were tested for antibacterial and antifungal activity. Potential of compound as antibacterial was assessed by less formazan production (Figure 3A). Based on IC_50_ and MIC determination, compound exhibited IC_50_ of about 20 μgml^−1^ against *E. faecalis*, and greater than 20 μgml^−1^ against *S. aureus* and *Aeromonas hydrophila*, as compound showed only 38.3% and 48% inhibition against *A. hydrophila* and *S. aureus* respectively (Figure 3B and Table 1). In antifungal activity, 10 μgμl^−1^ concentration of compound showed inhibition of *B. sorokiniana* (57.14%), *A. flavus* (52.5%), and *A. alternata* (91.25%) were observed as compared to control (Figures 4A,B and Table 2). No growth was observed in *P. drechsleri* at 1 μgμl^−1^. In addition to antifungal, Terrein had shown antioxidant activity by DPPH scavenging effect (Figure 5A). Compound exhibited moderate antioxidant activity with IC_50_ value 112 μgml^−1^ (Figure 5B).

**Table 1:** Representation of IC_50_ value of Terrein against bacterial pathogens *S. aureus, A. hydrophila, and E. Faecalis*.

**S.No**	**Bacterial pathogens**	**IC_50_ value**	**MIC**
1	*S. aureus*	>20 μgml^−1^	30 μgμl^−1^
2	*A. hydrophila*	>20 μgml^1^	40 μgμl^−1^
3	*E. Faecalis*	20 μgml^−1^	25 μgμl^−1^

**Table 2:** Reduction of fungal pathogens growth at 10 μgμl^−1^ treated with Terrein.

**S.no**	**Plant pathogenic fungi**	**Reduction % at 10 μgμl^−1^**
1	*Aspergillus flavus*	52.5
2	*Alternaria alternata*	91.25
3	*Bipolaris Sorokiniana*	57.14
4	*Phytophthora* sp	100%
5	*Fusarium oxysporum*	NIL

### Effect of extracts on viability and proliferation of cells

**Figure 6:**
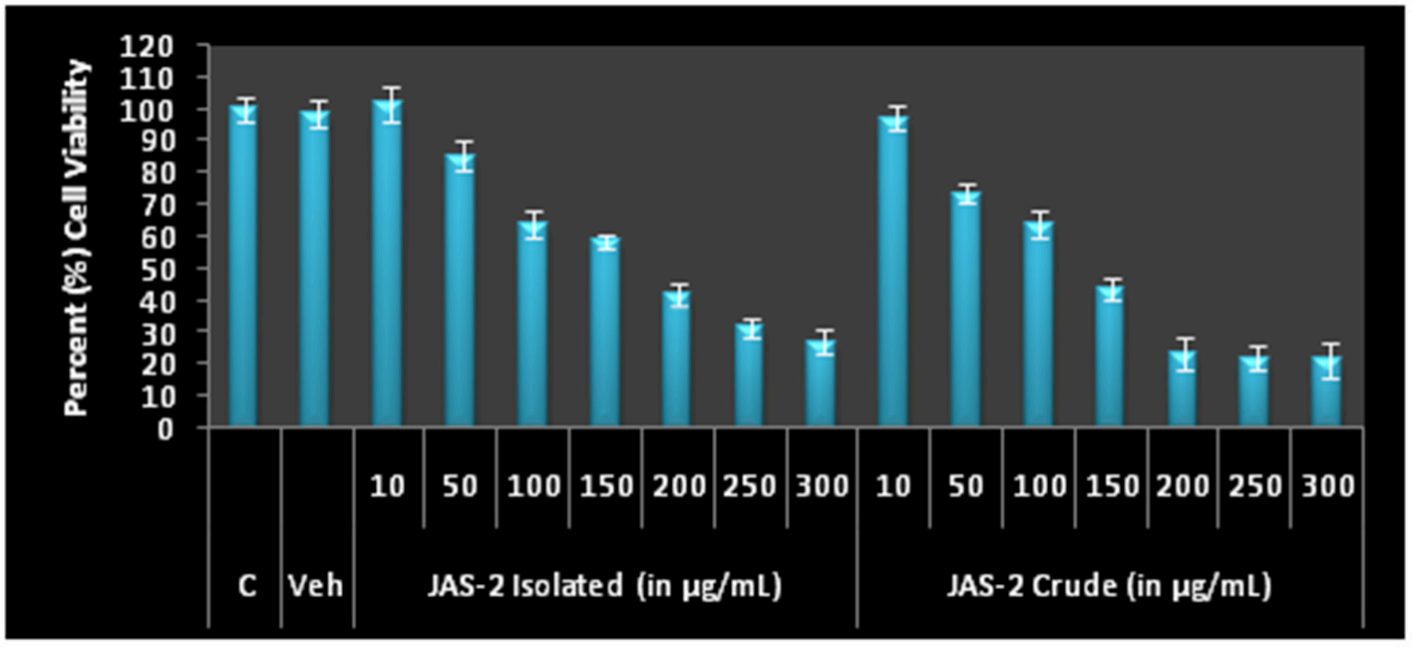
Cell viability and Antiproliferative profile of A-549 Lung cancer cells after treatment with different concentration of crude metabolites and Terrein for 24 h by MTT assay. The IC_50_ values of both extracts were determined on the basis of 50% O.D. as comparison to control.

The pure compound and crude extract were tested for cytotoxicity and anti-proliferative activity against lung cancer line (A-549). The crude of JAS-2 and pure compound exhibited significant toxicity and antiproliferation activity against A-549 cells. The IC_50_ values of pure and crude complexes are 170.99 ± 4.24 and 121.91 ± 4.82 μgml^−1^ respectively (Table 3) (Figure 6).

**Table 3:** Representation of IC_50_ value ± *SD* of compound against A-549 human lung cancer cell line (*n* = 4).

**Incubation time**	**A549-Human lung cancer line**	
	**IC_50_ value in μgml^−1^**	
	JAS-2 Pure form (Terrein)	JAS-2 Crude
24 h	170.99 ± 4.24	121.91 ± 4.821

### Analysis of cell/nucleus morphology with hoechst 33342/propidium iodide (PI) staining

**Figure 7:**
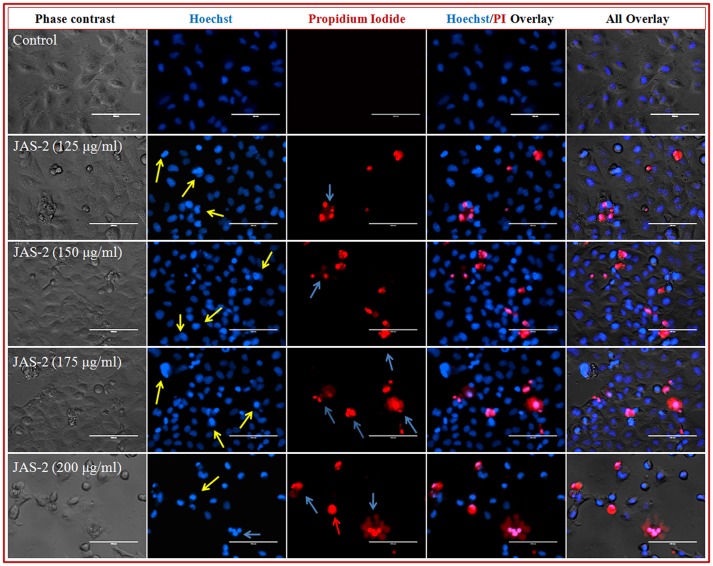
Representative grouped images showing morphological changes in nuclei of lung cancer (A-549) cells detected with dual staining of Hoechst 33342/PI. Cells were treated with different concentrations of pure compound for 24 h and imaged by inverted fluorescence microscope. Early apoptotic cells with yellow arrows, late apoptotic cells with fragmented nuclei indicated with blue arrows and necrotic cells with red fluorescent nuclei indicated with red arrows.

Chromatin condensation and fragmentation of nucleus was observed. The cells were stained and visualized by phase contrast and fluorescence microscopy. As the compound concentration increases, the nucleus condensation also increased (Figure 7). Consistent level of blue fluorescence nuclei were seen in control cells. Further, apoptotic cells in 3rd lane of figure was clearly visualized due to induction of cell death and cell membrane disruption. At very lower concentration (125 μgml^−1^) of compound apoptotic cells with deep blue fluorescent nuclei was seen, which are indicated with yellow arrow. However, at higher concentrations (200 μgml^−1^) late apoptotic cells with fragmented nuclei (indicated with blue arrow) and necrotic cells with red fluorescent nuclei (indicated with red arrow) were clearly observed (Figure 7). Increasing the frequency of fragmented nucleus in a dose dependent manner indicated that, compound favors apoptotic mode of cell death. 4 and 5th lane of Figure 7 shows Hoechst/PI and all overlays respectively.

### Effect of pure extract on distribution of cell cycle phases by FACS

**Figure 8:**
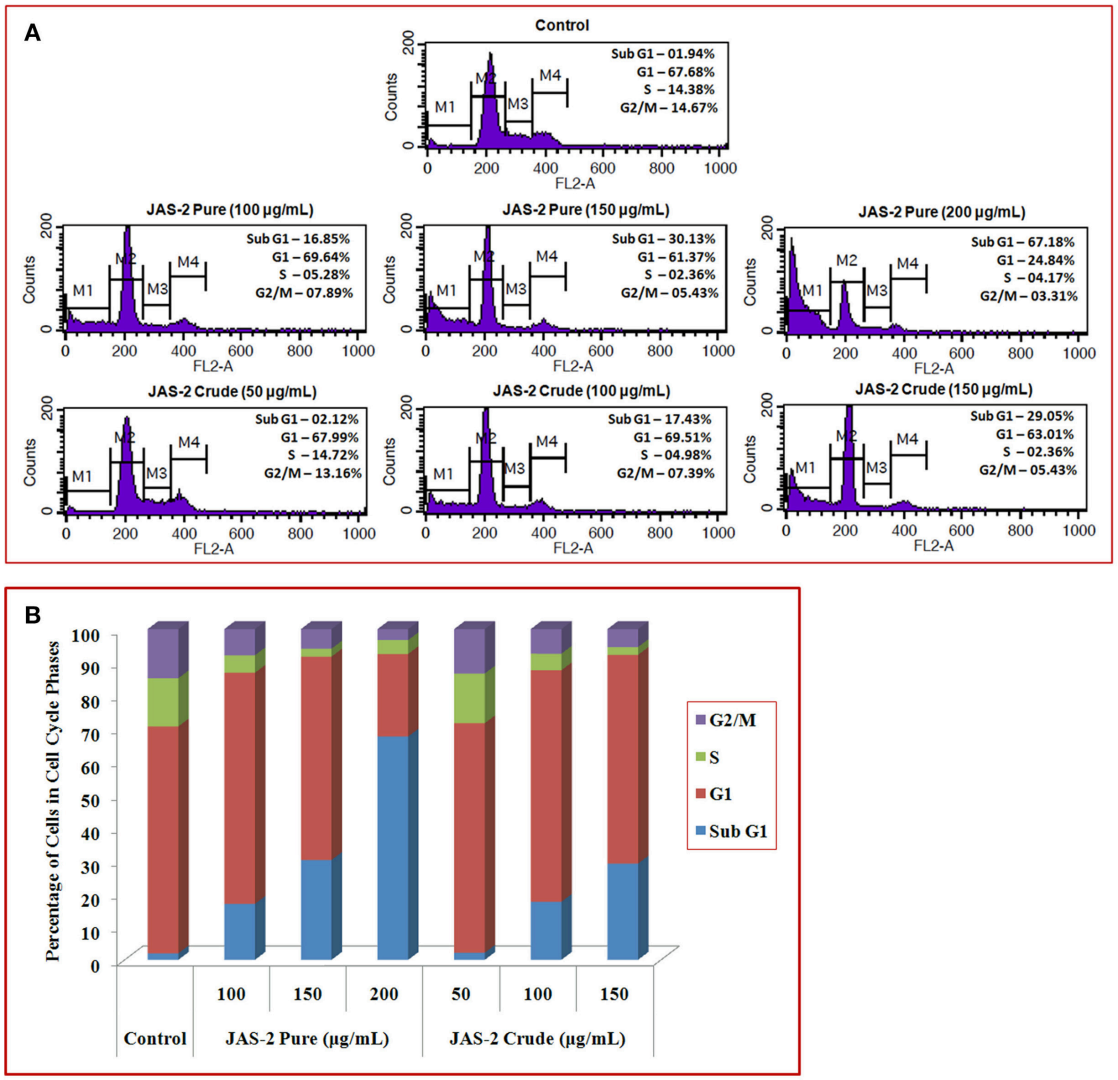
**(A)** Flow cytometry analysis of A-549 lung cancer cells of control and treated with crude and pure/isolated Terrein extract and cell cycle distributions (delay/arrest) were checked on the basis of DNA content during cell cycle phases. **(B)** Graphical presentation showing percent (%) of cells in different cell cycle phases. Histogram indicates that isolated Terrein and other extract were potentially induces apoptosis in A-549 lung cancer cells.

The ability of Terrein (pure extract) to induce cell death or persuaded cell cycle delay, FACS was done with PI staining. The cells were not arrested in any phase of cell cycle which indicates no cell cycle delay appeared due to extracts (either pure or crude) exposure (Figure 8A). However, maximum changes occurred in sub-G1 (apoptotic cell death) phases of all treated groups. In crude extract treated groups apoptotic cell death were significantly increased from control (2%) to higher concentration (29%) (150 μgml^−1^, crude extract) (Figure 8A). Whereas, the cells which were treated with Terrein (pure) extract, showing 16% (in lower concentration, 50 μgml^−1^) to 69% (in higher concentration 150 μgml^−1^). Histogram indicates (Figure 8B) that pure/isolated Terrein extract was potentially induces apoptosis in A-549 lung cancer cells.

## Discussions

This study deals with isolation and characterization of compounds endowed with multiple potentials extracted from endophytic fungus associated with *Achyranthus aspera*. Goutam et al. (2016b) the pure compound was identified as 4, 5-Dihydroxy-3-(1-propenyl)-2-cyclopenten-1-one and the trade name of this compound is Terrein. To the best of our knowledge this is first report which has shown isolation of Terrein from fungus *A. terreus* which is endophytic in nature. The compound has been isolated from various strains of fungi such as *Microspora* (Grove, 1954) *Pencillium Phoma* (Dunn et al., 1975) and reported to have anticancer activity against different cell lines. Secondly innumerable work has been carried out toward identification and characterization of biological active compounds from endophytic fungi which could serve as basic template in combinatorial chemistry. Mousa and Raizada (2013) reviewed diverse class of secondary metabolites with their antimicrobial perspective. Among endophytic metabolites, Taxol is a multibillionaire antineoplastic drug which has been studied extensively. Endophytes isolated from medicinal plants possess strong fungicidal, bactericidal, and cytotoxic metabolites (Wang et al., 2007). Recently Gressler et al. (2015) reported that compound Terrein has suppressed the growth of pathogens *Fusarium graminearum* and *Aspergillus fumigatus*. Terrein also exhibited anti-oxidant activity as it has scavenged 50% DPPH at 112 μgml^−1^. The antioxidant property can be correlated with earlier reports as melanogenesis inhibitor (Park et al., 2004; Kim et al., 2007). It was also found that Terrein inhibits age-related inflammation by promoting an antioxidant response in aged human diploid fibroblast (HDF) cells (Lee et al., 2015).

The compound has been tested against different types of cancer cell lines such as pancreatic cancer, breast cancer (Liao et al., 2012), human cervical cancer (Porameesanaporn et al., 2013) skin cancer, and prostate cancer (Arakawa et al., 2008). Among the different types of cancer identified globally, lung cancer is found to be the most prevalent type (12.7% of 1.61 million) (Ferlay et al., 2008). This persuaded us to study anticancer property of Terrein against human lung cancer cell line. This is the first study to Terrein the compound against lung cancer.

Pure compound had shown lower toxicity (higher IC_50_ value) than crude extract against A-549 cells (Figure 6 and Table 1). This could be due to the presence of compound other than Terrein which simultaneously aids in anticancer activity (Figure 6) (Tables 4, 5). In past 40 years, natural products played significant role as cancer chemotherapeutic agents, either in their unmodified (natural form) or synthetic forms (Kinghorn, 2008). Among plant derived products, many antitumor compounds have been reported from medicinal plants such as, bisindole (vinca) alkaloids, the camptothecins, the epipodophyllotoxins, and the taxanes (Berger and Benjamin, 2004). Among biological diverse organism endophytes produces a number of significant compound. Among which most of them are having extreme clinical use. Hence it is important to explore the unexplored flora to identify newer molecules.

**Table 4:** Gas chromatography mass spectroscopy (GCMS) analysis of pure fraction separated from CC (Terrein).

**Peak**	**R.T**	**Area (%)**	**Name of compounds**
1	10.090	100.00	Terrein
2		100.00	

**Table 5:** Profile of chemical compounds from GCMS analysis of crude metabolites of selected strain (JAS-2).

**Peak**	**R.T**	**Area (%)**	**Name of compounds**
1	5.589	1.33	Borane, tributyl-
2	5.705	0.09	2-HEXANONE, 3-METHYL-4-METHYLENE-
3	5.810	0.11	3H-Cyclopenta[c]pyridazin-3-one, 2,5,6,7-tetrahydro-
4	6.708	0.18	7-(BROMOMETHYL)-7-PENTADECENE
5	6.782	0.09	2-Methyl-3(2-furyl) acrolein
6	6.884	0.27	1-Allylcyclopropanecarboxylic acid
7	7.063	0.17	2-PROPYL-2-PENTENAL
8	7.473	0.34	1-OXASPIRO[2.5]OCTAN-4-ONE, 2,2-DIMETHYL-
9	7.621	0.88	4-OCTEN-3-ONE, 4,6-DIMETHYL-, (E)-
10	7.781	0.67	2-Heptenal, 2-propyl-
11	9.098	3.23	Terrein
12	9.454	0.17	CYCLOPENTANONE, 3,4-BIS(METHYLENE)-
13	10.891	91.85	Terrein
14	14.635	0.63	1,2-Benzenedicarboxylic acid, bis(2-methylpropyl) ester
		100	

## Author contributions

JG was the principle investigator who designed the study, purified the compound isolated from endophytic fungi JAS-2 and characterized for antibacterial, antifungal antioxidant activity. AM and VT characterized the compound. GS and BK accessed the anticancer activity of compound. All authors read and approved the final manuscript. RK provided reagent material and analytical tools. VR helped in finalizing the manuscript.

### Conflict of interest statement

The authors declare that the research was conducted in the absence of any commercial or financial relationships that could be construed as a potential conflict of interest. The reviewer BS declared a shared affiliation, though no other collaboration, with the authors JG, GS, VT, AM, RK and BK to the handling editor, who ensured that the process nevertheless met the standards of a fair and objective review.

## Supplementary material

Figure S1 GCMS chromatogram showing profile of **(A)** pure compound (Terrein) and **(B)** crude extract of JAS-2.
