# Supplementary figures and images for: Isolation and Characterization of “Terrein” an Antimicrobial and Antitumor Compound from Endophytic Fungus Aspergillus terreus (JAS-2) Associated from Achyranthus aspera Varanasi, India
Source: Front Microbiol. 2017 Jul 25;8:1334. doi: 10.3389/fmicb.2017.01334 (PMC5526331; doi:10.3389/fmicb.2017.01334)

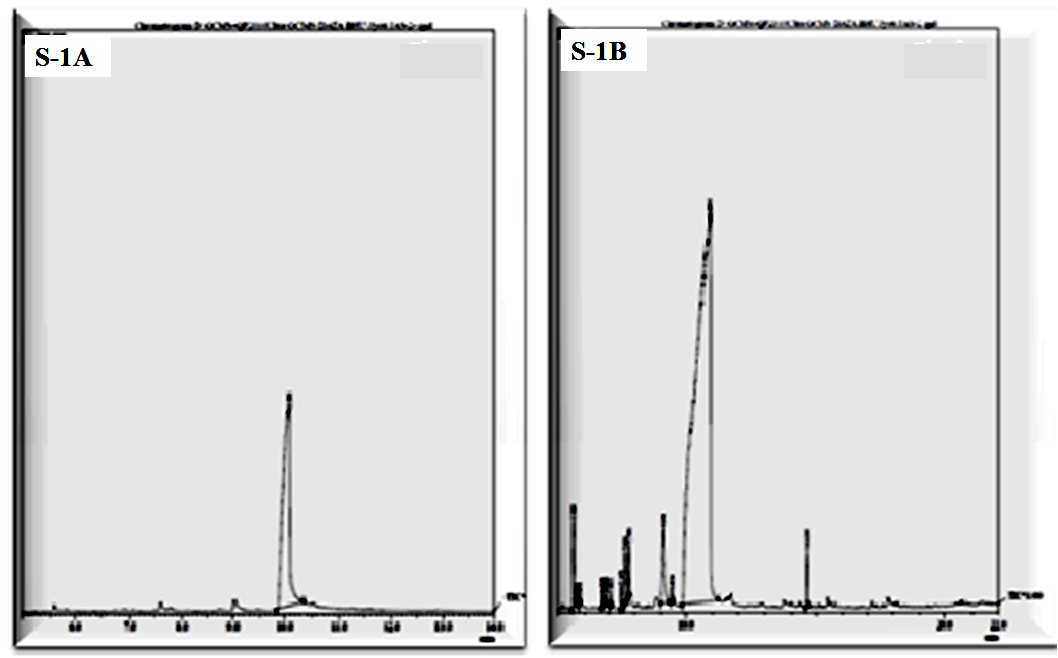

Supplement: Figure S1 — GCMS chromatogram showing profile of (A) pure compound (Terrein) and (B) crude extract of JAS-2. [file Image1.TIF]
